# Measuring the Influence of Environmental Conditions on Automotive Lidar Sensors

**DOI:** 10.3390/s22145266

**Published:** 2022-07-14

**Authors:** Clemens Linnhoff, Kristof Hofrichter, Lukas Elster, Philipp Rosenberger, Hermann Winner

**Affiliations:** Institute of Automotive Engineering, Technical University of Darmstadt, 64289 Darmstadt, Germany; kristof.hofrichter@stud.tu-darmstadt.de (K.H.); lukas.elster@tu-darmstadt.de (L.E.); philipp.rosenberger@tu-darmstadt.de (P.R.); hermann.winner@tu-darmstadt.de (H.W.)

**Keywords:** automated driving, lidar, model, fog, rain, snow, sun, environment, simulation

## Abstract

Safety validation of automated driving functions is a major challenge that is partly tackled by means of simulation-based testing. The virtual validation approach always entails the modeling of automotive perception sensors and their environment. In the real world, these sensors are exposed to adverse influences by environmental conditions such as rain, fog, snow, etc. Therefore, such influences need to be reflected in the simulation models. In this publication, a novel data set is introduced and analyzed. This data set contains lidar data with synchronized reference measurements of weather conditions from a stationary long-term experiment. Recorded weather conditions comprise fog, rain, snow, and direct sunlight. The data are analyzed by pairing lidar values, such as the number of detections in the atmosphere, with weather parameters such as rain rate in mm/h. This results in expectation values, which can directly be utilized for stochastic modeling or model calibration and validation. The results show vast differences in the number of atmospheric detections, range distribution, and attenuation between the different sensors of the data set.

## 1. Introduction

The development and validation of automated driving functions rely more and more on virtual testing methods. As the interface between the vehicle and the environment, modeling perception sensors plays a major role in simulation-based development. Radars, lidars, and cameras not only perceive objects in their surroundings, but are also influenced by environmental conditions due to weather. Previous research, e.g., [1,2,3,4,5], has already identified a strong influence of rain, snow, fog, etc., on automotive lidar sensors. These publications also introduce theoretical physical models for the impact of the environmental conditions on lidar. Apart from the physics of the interaction of infrared light beams with hydrometeors and particles in the atmosphere, there are several design parameters and signal processing algorithms only known to the sensor manufacturer. For modeling an existing lidar sensor by a third party, stochastic elements have to be introduced and certain parameters such as dynamic power levels or thresholds need to be calibrated. Furthermore, the model as a whole, including physics, hardware, and signal processing, needs to be validated. Therefore, there is a demand for a data set recorded with automotive lidar sensors and quantifiable weather reference, covering a wide range of environmental influences.

The state of the art of data sets with environmental influences on lidar sensors is divided into two categories. The first category contains measurements performed in artificial weather conditions. Rasshofer et al. [1] developed mathematical models for the influence of rain, fog, and snow on lasers for physical modeling with validation tests in artificial rain. However, they did not test automotive lidars with an included signal processing. Tests with actual lidar sensors in weather chambers were performed in [6,7,8,9,10,11,12]. Outdoor devices to generate synthetic rain where developed in [13,14,15]. The major benefits of these artificial weather conditions are controllability and accessibility. However, the problem with using artificial weather conditions is that they have to be validated themselves first. As stated in [10], that is not always the case. In their lidar measurements in a weather chamber, “rain pillars” are clearly visible underneath the nozzles of the rain generator and it is also not guaranteed that drop size distributions match with real conditions. Furthermore, in a chamber it is not possible to measure the influence in the atmosphere without a “hard object” in the background as there are always walls surrounding the sensor. Additionally, the intensity of the conditions e.g., in case of [7] is set to extreme values. Very dense fog is used, which would most likely be excluded in the Operational Design Domain (ODD) of a level 3 or level 4 automated vehicle, just like other intense weather conditions according to [16], while light fog also has an impact on the sensors, as measurements in this publication will show.

The second category in the state of the art is comprised of measurements performed in real-world weather conditions. In this category, dynamic data sets exist recorded with test vehicles on the road in different weather conditions, such as nuScenes [17], SeeingThroughFog [18], and RADIATE [19]. All three data sets contain multiple different perception sensors and a limited set of weather conditions. However, they come only with weather labels or descriptions rather than quantified reference data for rain rate or visibility in fog. Additionally, the dynamic nature of the data sets does not allow for direct comparison of influences in different conditions. For this purpose, stationary setups are more suited. An early example of such a stationary setup is proposed by Grantham et al. [20]. A military lidar is used for data recording in rain and fog providing variations in target intensity and false echos depending on synchronized rain intensity and visibility measurements. However, lidar development has come a long way since that publication and automotive lidars differ from military sensors in terms of power and range. Filgueira et al. [21] presented a stationary setup with a Velodyne lidar on the side of a road. This setup allows for material comparisons of different objects of interest, such as asphalt, concrete, stone, and retro reflectors. The data comprises five rain events with five different rain intensities ranging from 0 to 8 mm/h over a period of 2 months. The detections on the different materials are analyzed in terms of range, intensity, and the number of points on the objects of interest. Vargas Rivero et al. [22] took a similar approach mounting a Valeo Scala lidar on the top of a roof, oriented downwards onto an asphalt parking lot. Measurements were taken over a period of 9 months covering rain, snow, fog, and different background lighting conditions. As a reference, a weather station at a distance of 7 km is used to provide rain intensities in 5 min intervals and snow and fog data in 1 h intervals. This data set is intended for the development of weather classification with lidar rather than model calibration and validation, where the comparably low reference frequency would not be sufficient due to rapid changes in precipitation intensity and visibility. The most recent publication by Wichmann et al. [23] also considers a stationary setup. They utilized a self-developed one-channel direct time-of-flight lidar in two configurations. The first one is a quasi mono-static setup with a retroreflector and the second one is a bi-static setup with a receiver at 100 m distance from the transmitter. Thies Clima sensors are used as synchronized references directly at the site of the experiment. Measurements were conducted over one year, covering the weather events of rain, snow, fog, graupel, and hail. The analysis provides extinction coefficients and backscattering strengths over different precipitation intensities, which are very valuable for modeling the physical aspects of lidar simulations. While this publication gives a very promising insight into the time domain and actual amplitudes at the receiver diode, there is still one step further in simulating actual automotive lidar sensors. That is the signal processing with peak detection and dynamic thresholding, which is different for every lidar system.

**Figure 1:**
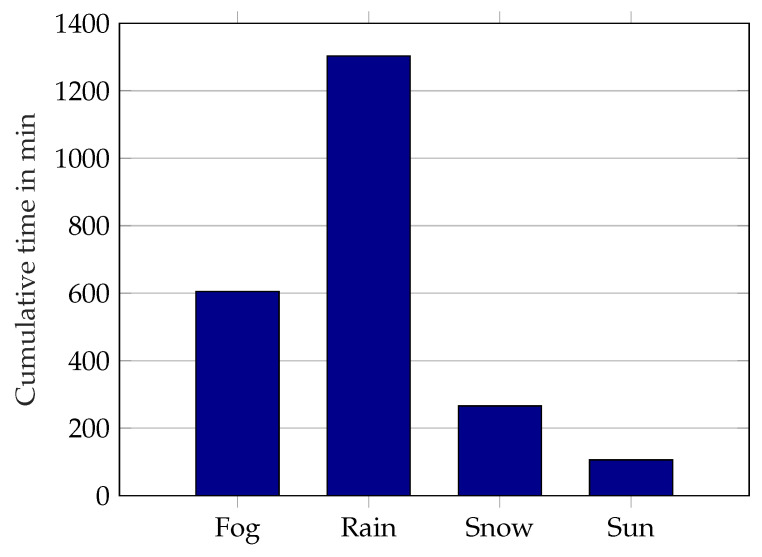
Time coverage of weather categories in the data set.

In this publication, a novel data set is introduced, explicitly designed to quantify the influence of rain, fog, snow, and sunlight for stochastic modeling as well as model calibration and validation. Measurements were taken with three different lidar sensors over a period of 6 months in a stationary setup with reference targets at different distances. The time coverage of the different weather conditions in the data set is depicted in Figure 1. The time coverage does not represent the real-world occurrence of the conditions, e.g. the sun measurements were taken manually because of dependencies on cloud formations. Therefore there are fewer sun measurements than other categories and a few nighttime measurements for comparison were also added manually. In addition to the lidar sensors, weather sensor data are recorded to provide time-synchronized reference data for rain intensity, meteorological visibility, and sunlight brightness. To the knowledge of the authors, there is no other data set with automotive lidars containing rain, fog, snow, and direct sunlight in a repeatable stationary setup, as also stated in [24] (Table 6). The design of the experiment is targeted to create calibration and validation data of influences by hydrometeors during signal propagation and influences by sunlight. Influences directly on the sensor cover are excluded, which coincides with the definition of an environmental condition by Goelles et al. [25]. The data set is analyzed to provide parameters that can be directly used for model calibration and validation of models of the recorded lidar sensors, such as the number of detections caused by rain at a certain rain rate.

## 2. Experiments

In order to analyze the influences of precipitation and sunlight on different active perception sensors, a long-term experiment is set up. Data are recorded for a period of 6 months from October 2021 to March 2022 on the August-Euler Airfield in Darmstadt, Germany. The setup consists of a test station with perception sensors and reference sensors for environmental conditions as well as reference targets. The test station is oriented 160° with respect to north. The test area is approximated 109 m above sea level. The analysis of the measurements in Matlab yielded expectation values for modeling, calibration, and model validation.

### 2.1. Data Recording

The station consists of a waterproof housing for the power supply and a measurement computer. On the outside, different sensors are mounted in a modular design. The perception sensors are mounted 0.4–0.6 m above the ground. The exact mounting positions are set in the “/tf_static” topic in the recordings. For the experiments, the following perception sensors with their respective configuration were connected to the station and included in the data set:

- Blickfeld Cube 1 Lidar, 64 layers, 72° × 20°, 8.4 Hz, 3× multi-echo,
- Ouster OS1 32 Lidar, 32 layers, 360° × 45°, 10 Hz, strongest return,
- Velodyne VLP16 Lidar, 16 layers, 360° × 30°, 10 Hz, strongest return,
- Aptiv ESR 2.5 Radar (not used in this work).

Furthermore, three reference sensors for the environmental conditions are connected and part of the data set:

- OFS Fog Sensor (Optical sensors ONED 250),
- Thies Clima US NHTFB 4.9200.00.000 Weather Sensor,
- Mini PWS Fog and Precipitation Sensor (not used in this work).

**Figure 2:**
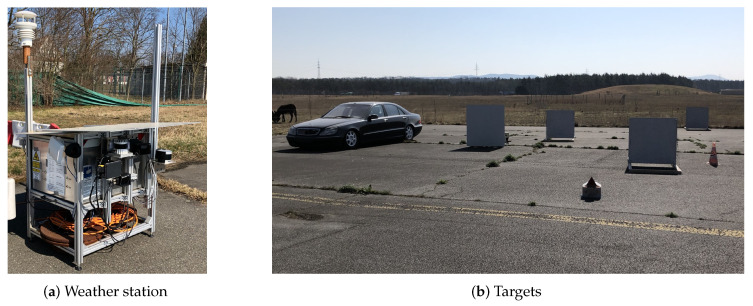
Stationary measurement setup for data set generation.

Additionally, a camera as a reference is part of the station. The setup is depicted in Figure 2a. Opposite the station reference, targets for lidar and radar are placed. As a calibration target for radar, which is not covered by this publication, a Cube Corner Reflector (CCR) is used. For lidar, targets with a reflectivity of 50% are located at 50 m, 58 m, and 66 m distance. These targets are plywood boards painted with RAL7047 lacquer. The reflectivity was validated against a reference material with the procedure described in [26]. Additionally, one more lidar target with undefined reflectivity is located at 55 m and a stationary vehicle is placed as an extended target, as shown in Figure 2b. The latter are not used in this publication.

The perception sensors measure all output detection data i.e., point clouds with locations and intensities. The three reference sensors output the following measurement quantities:

- precipitation rate in mm/h,
- ambient temperature in °C,
- relative humidity in %,
- air pressure in hPa,
- wind velocity (incl. direction) in m/s,
- brightness in lx with direction of light source,
- synop present weather code [27] for classification,
- meteorological visibility in m.

The OFS used for measuring the meteorological visibility outputs one data point per minute as a mean of that time frame. The Clima sensor used for all other reference values outputs a new data point every three seconds as a mean value of the last 10 s. This time shift is corrected along with other delays in the analysis. The reference sensors and the perception sensors are all connected to a measurement computer running the Robot Operating System (ROS) on Ubuntu 18. A ROS node uses the values of precipitation rate, synop code, and meteorological visibility as triggers to automatically start recordings at defined thresholds of the parameters. The synop code gives classifications of the present weather and is used to identify snow. Precipitation rate and visibility each have multiple thresholds. Once one threshold is exceeded, data are recorded for 10 min. Another recording is only started when the next higher threshold is exceeded. The threshold limits are reset, once a value has fallen below the previous threshold. This form of hysteresis prevents recordings from starting right after the previous recording has stopped, reducing the total amount of data.

### 2.2. Analysis Methodology

In a first step, all measurements are sorted into four categories: fog, rain, snow, and direct sunlight. Rain and snow are separated by the synop code and fog by low visibility with a non-precipitation synop. Sun measurements were taken manually when sun influence was visible in the live data feed and sorted directly. By checking the intensity of detections between the station and the target area in the recordings, a flag for possible obstruction by objects in the field of view is set. Flagged measurements are inspected manually and sorted out in case of actual obstruction.

For every category, parameters of the present environmental condition are paired with data from the sensor detections over all measurements within that category, e.g., the rain rate in mm/h with the number of detections from a lidar sensor in the atmosphere. There are three kinds of regions of interest for sensor detections. The first one is the atmosphere, where under clear conditions no detections are generated. Detections in this region can only originate from precipitation, fog, or sun influence. To analyze atmospheric detections a filter is set up between −36° and 36° azimuth, so all sensors have the same horizontal angle area, and for elevation only positive angles are applied to filter out detections from the ground. The maximum distance for the filter is set to 30 m to retain a safe distance to the start of the target region. The Blickfeld lidar sends 5760 beams into the region and the Ouster lidar 5325. The Velodyne lidar has 2880 beams entering the atmospheric filter region. The second kind of region comprises each individual sensor target. A Cartesian filter is applied for all three sensors for the individual targets. The third region is the pavement, however, because the pavement is quite uneven and preliminary experiments with a water film sensor on one position were inconclusive, this region of interest is neglected in this analysis. However, there is one more influence. Water or snow directly on the sensor cover might manipulate the expectation values as it adds an attenuation, not quantified by any reference sensor. This is counteracted with a roof extension on the measurement station over the perception sensors. However, with strong winds, this will potentially still be an issue.

**Figure 3:**
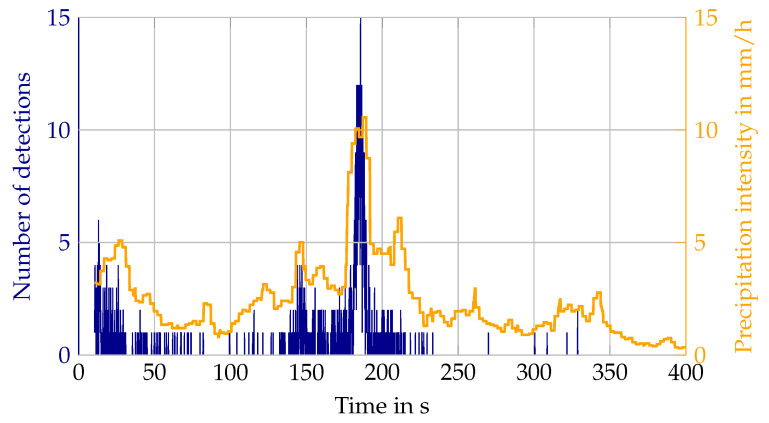
Example of a rapid change in rain intensity.

As for lidar values, mainly the relative number of detections and the range distribution are used to quantify the environmental impact. The relative number of detections is the detection count within a previously defined filter region, divided by the total number of beams entering that region. This results in the detection probability per beam in the atmosphere caused by an environmental condition. Similar filtering is applied to the targets to give a detection probability for one target in certain conditions. These values describe the existence and state, in the form of the distance of the lidar detection, and constitute the first step for simulation to answer the questions, how many detections need to be added due to the environmental condition and where do they need to be added. At this point, no distinction between layers or elevation angles is made and the intensity values of the detections are disregarded. Both will be addressed in future work. With the different distances of the lidar targets, a detection range is derived from the number of detections. Detection ranges describe the attenuating influence of the environmental condition on the detection of other objects. These values are therefore the minimum set to parameterize a stochastic simulation model or validate a physical model. For fog, the sensor values are compared to the visibility in m, for rain and snow to the precipitation rate in mm/h, and for the sun to the brightness in lux. In the simulation, these parameters for the environmental conditions are sometimes grouped in classes, e.g., light rain, moderate rain, and strong rain, for example in the current version of the Open Simulation Interface [28] (OSI). However, Figure 3 shows that environmental conditions change rather dynamically. In this example the rain rate changes from 4 mm/h to over 10 mm/h and back in just 20 s. As the detection count of the Velodyne lidar follows this change, it needs to be represented as a ground truth parameter in the simulation. Nevertheless, to calculate estimation values as means, the sensor values need to be sorted into bins. For precipitation rate and visibility in fog, a logarithmic scale with 25 bins per decade is set. This bin discretization was found as a compromise between binning as fine as possible and making sure that bins contain multiple data points for calculating the mean value.

## 3. Results

The analysis is sectioned into the categories of fog, rain, snow, and sunlight. For each category, the results from the described methodology are displayed in plots each combining a sensor value with a reference value.

### 3.1. Fog

**Figure 4:**
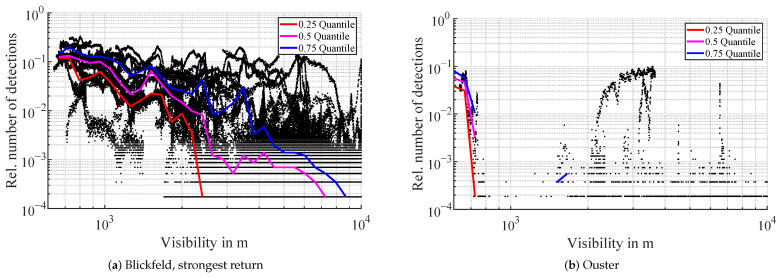
Relative number of detections in the atmosphere in foggy conditions, scaled to the total number of beams entering the atmospheric filter region. Every black dot represents one scan of a lidar i.e., one time step. The colored lines are the quantiles for each logarithmic visibility bin.

As a first value, the existence of detections in the atmosphere is analyzed. In Figure 4 the relative number of detections in the atmospheric filter region is graphed over the measured visibility. The number of detections is scaled to the number of beams entering the spherical filter region giving an expectation value for the existence of a detection in a single beam. The filter region is covered by 2880 beams of the Velodyne lidar, 5760 beams of the Blickfeld lidar, and 5325 beams of the Ouster lidar.

Apart from a few outliers, the detection count of the Velodyne lidar remains 0 throughout the entire visibility range, therefore, no plot is displayed. For the Blickfeld lidar, the number of detections of the strongest return starts to rise when the visibility falls under 2500 m (Figure 4). The Ouster lidar starts to show atmospheric detections below a visibility of 800 m. The plot of the Blickfeld lidar shows a lot of outliers, which are not random noise but follow distinct curves. Linear interpolation of the visibility values from the OFS sensor is a possible explanation and will be analyzed in more detail in the discussion section.

Furthermore, the Blickfeld sensor is multi-echo capable, so it is able to generate more than one detection per beam. However, only a few second return detections are generated. They are starting to appear under 1000 m visibility and rise to a median of 0.001 relative detections in the atmosphere in the lowest visibility bin.

**Figure 5:**
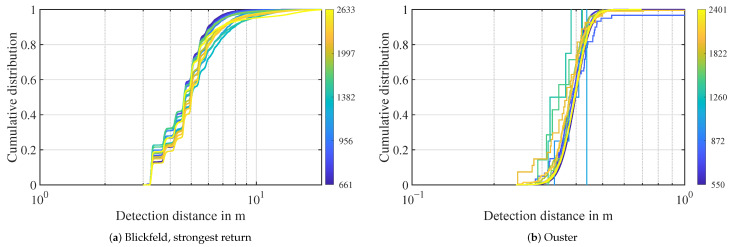
Distance distribution of atmospheric detections in fog. The visibility in m is denoted as color, each line represents a logarithmic visibility bin. Note the different scaling on the distance axis.

Figure 5 depicts the Cumulative Distribution Function (CDF) of the distances of the detections in the atmosphere during fog. Every line represents the detections in one visibility bin. The color denotes the visibility in m. As the Velodyne sensor does not generate atmospheric detections in fog, only Blickfeld and Ouster lidars are analyzed. For the Blickfeld, 95% of first return detections are located within a distance of 3 m and 6.5 m to 10 m, depending on the visibility. Detections below 3 m are filtered out by the sensor. In general, detections in lower visibilities (blue lines) are closer to the sensor. Another noticeable feature is the waviness in the CDFs. At lower distances, there are distinct steps in all visibilities. The step size gradually decreases with distance. According to the manufacturer, this is due to a sinusoidal sensitivity of the receiver in the lidar sensor. In the Ouster lidar, atmospheric detections in fog are very close to the sensor. Except for the lowest visibility bin, 95% of detections are within 0.2 m and 0.5 m in front of the sensor.

**Figure 6:**
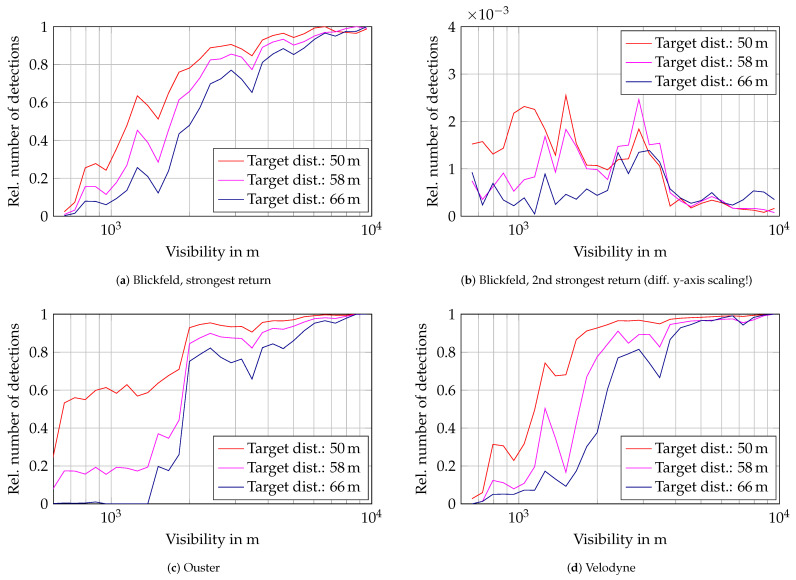
Relative number of detections on three 50% targets over visibility. The number of detections is the mean value per visibility bin scaled to the respective maximum detection count on the targets in clear conditions.

The detection range in fog is assessed with three targets with 50% reflectivity. The relative number of detections on these targets is depicted in Figure 6 over the visibility in m. The detection count on the targets is scaled to the maximum number of (first return) detections in clear conditions to get a detection probability per beam, similar to the previous analysis of the number of detections in the atmosphere. For all three sensors, the detection probability decreases slightly with decreasing visibility and drops rapidly at around 2000 m. In Figure 6b the second strongest return of the Blickfeld lidar on the targets is shown. The detection count rises slightly below 4000 m visibility, but only has detection probabilities under 0.3%. The general trends of the relative number of detections on the targets give guidance for the modeling of attenuation due to fog. Characteristic values such as the 50% mark at the respective distances can be used to calibrate detection thresholds in the models.

### 3.2. Rain

The same analysis for fog is applied to rain, starting with the relative number of detections in the atmosphere compared to the precipitation rate in mm/h.

**Figure 7:**
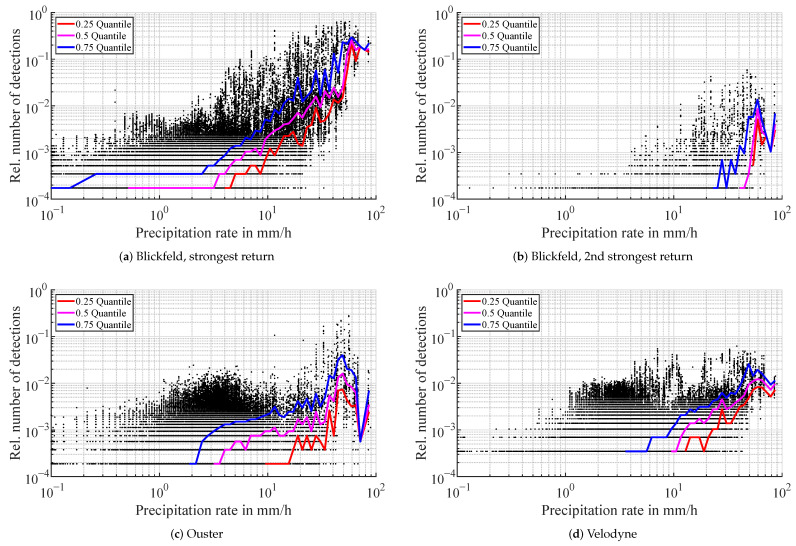
Relative number of detections in the atmosphere in rainy conditions. The number of detections is scaled to the total number of beams entering the spherical filter region for the detection count. Every black dot represents one scan of a lidar i.e., one time step. The colored lines are the quantiles for each logarithmic precipitation rate bin.

The Blickfeld lidar generates first echo detections with a median starting to increase at around 3 mm/h as can be seen in Figure 7a. The relative number of detections in the atmosphere shows a growing trend over the rain intensity up to over 0.2 at 59 mm/h. At higher rain rates, second returns are also generated with a median starting to rise at around 45 mm/h, see Figure 7b. Few third returns exist and are negligible compared to first and second returns. In contrast to the fog measurements, in rainy conditions, the Velodyne lidar does output detections in the atmosphere, as visualized in Figure 7d. The median number of detections rises over the precipitation rate reaching a maximum median detection probability per beam of around 0.0015. Individual detection probabilities however reach values of up to 0.06. The trend of detection probabilities of the Ouster lidar (Figure 7c) is comparable in magnitude to the Velodyne sensor. In all three sensors, there are outliers. This phenomenon is analyzed in more detail in the discussion section. The volume of outliers at lower rain rates is due to the larger sample number and also the logarithmic binning.

**Figure 8:**
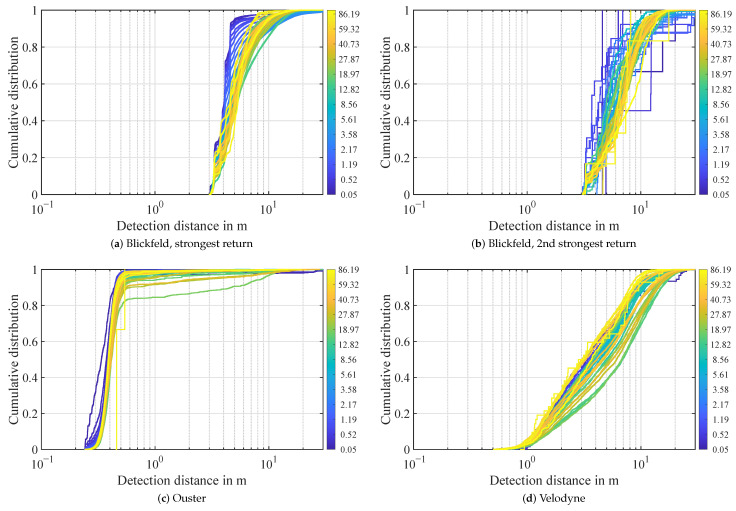
Distance distribution of atmospheric detections in rain. Every line represents the distribution in a logarithmic precipitation rate bin. The rain rate in mm/h is denoted as color.

The distance distribution of detections in the atmosphere during rain is depicted in Figure 8. The distance to 95% of first return detections in the atmosphere from the Blickfeld lidar ranges from 3 m to 14 m. There is a trend of detections registered closer to the sensor for lower rain rates and further away for stronger precipitation. Second return detections appear at similar distance distributions, as can be seen in Figure 8b. Similar to foggy conditions, the detection count of the Ouster lidar rises steeply in the range of 0.2 to 0.5 m. However, in rain, the detection distance goes further with the 95% mark passed at 2.5 m except for one outlier, where the range is stretched to over 8 m. For the Velodyne lidar, the distance to 95% of detections in the atmosphere ranges from 0.5 m to 18 m. Variations in the distance distribution between different precipitation intensities exist in the Velodyne lidar too, but there is no directly discernible correlation. A possible trend would be that in low rain rates, the detections are closer to the sensor, in medium rain rates further away and in strong rain close to the sensor again. This will be analyzed in future work.

**Figure 9:**
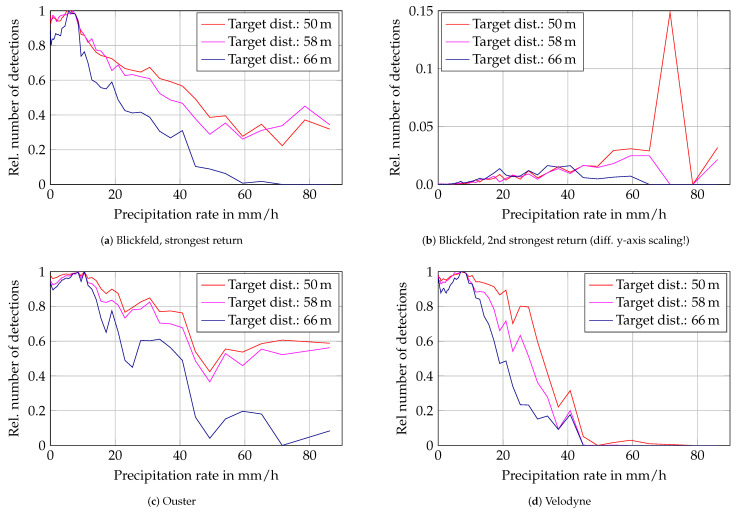
Relative number of detections on three 50% targets over rain rate. The number of detections is the mean value per precipitation rate bin scaled to the respective maximum detection count on the targets in clear conditions.

Detection probability on the targets starts to drop at around 10 mm/h as depicted in Figure 9 for all three lidar sensors. The Velodyne sensor loses detection of all targets at around 50 mm/h while the Blickfeld and Ouster lidars uphold detections on the 50 m and 58 m targets at all times. Only the 66 m target is lost at around 70 mm/h. It is interesting to note that the first two targets perform very similarly in the Blickfeld and Ouster lidars while the trend of the last target differs, although the distance between the first and second as well as the second and third target is the same. The Blickfeld lidar’s second return detection count rises gradually with increasing rain intensity, but stays comparably low. Except for one outlier, the relative number of detections on the targets stays below 0.04 for all targets.

### 3.3. Snow

**Figure 10:**
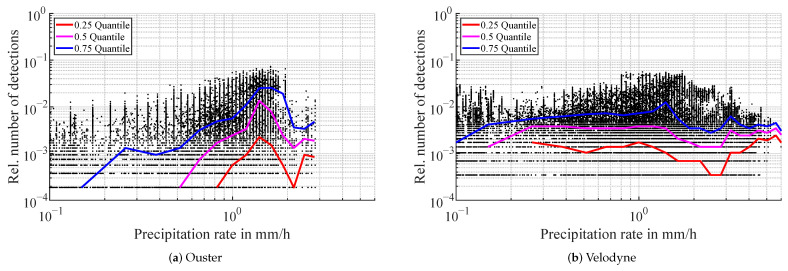
Relative number of detections in the atmosphere in snowy conditions. The number of detections is scaled to the total number of beams entering the spherical filter region for the detection count. Every black dot represents one scan of a lidar i.e., one time step. The colored lines are the quantiles for each logarithmic precipitation rate bin.

The relative number of atmospheric detections during snowfall is depicted in Figure 10. The filter region is the same as for fog and rain, so the relative detection count in the atmosphere i.e., the detection probability per beam is comparable. Unfortunately during two of the three recorded snow events, the Blickfeld sensor was not operational due to an unrelated software issue. Therefore, this section only contains results from the Velodyne and Ouster lidars. The Ouster sensor was operational during two snow events, limiting comparability over all measurements. However, single measurement comparisons are still viable, which is a better approach for snow anyways due to the following reason.

**Figure 11:**
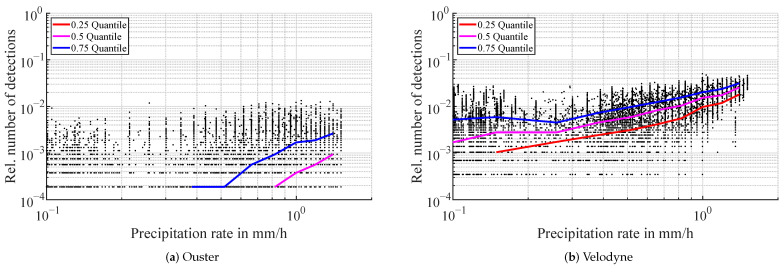
Relative number of detections in the atmosphere in snowy conditions from one day. The number of detections is scaled to the total number of beams entering the spherical filter region for the detection count. Every black dot represents one scan of a lidar i.e., one time step. The colored lines are the quantiles for each logarithmic precipitation rate bin.

In the aggregated plots over all snow measurements, there are a lot of outliers surpassing the mean values by an order of magnitudes. Additionally, lower median numbers of detection at higher precipitation intensities are quite counter-intuitive. Looking at individual measurements it becomes apparent that the scale between the values is quite different on different days. There are multiple possible explanations for this observation. The first one is that in one measurement there was snow built up on the sensor leading to an attenuation of the signal and consequently to a lower detection count. The other explanation is that snow is not equal to snow. Intensity is a combination of the number of snowflakes, their velocity, and their volume. Different combinations might have different effects on the sensor data. Additionally, there are hybrid forms of snow and other forms of precipitation, e.g., sleet. When only measurements from one day are considered, the correlation between number of detections and precipitation rate over all measurements from that day is much clearer, as depicted in Figure 11. How to distinguish between the different variations in snow and how to filter out measurements with attenuation due to moisture on the sensor cover has to be evaluated in future work.

**Figure 12:**
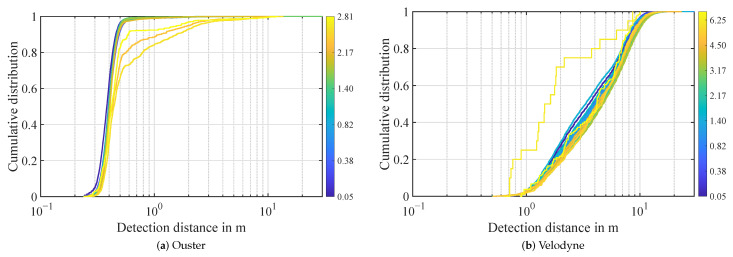
Distance distribution of atmospheric detections in snow. Every line represents the distribution in a logarithmic precipitation rate bin. The intensity in mm/h is denoted as color.

The distance distribution however does not seem to be affected by different kinds of snow or attenuation by snow buildup, as shown in Figure 12. In the Velodyne point cloud, 95% of atmospheric detections in snow are distributed over a range of 0.5 m to 11 m. As for the other conditions, the Ouster lidar has quite a narrow influence range of 0.2 m to 0.5 m of 95% of detections in snow under 2 mm/h. Above this snow rate, detections are spread out further up to 2.5 m. These distributions are more similar to rain.

The number of detections on the targets is not noticeably affected by snow in the measured precipitation rates. The mean number of detections registered by the Velodyne lidar on the last target decreases by a maximum of 6% at 0.4 mm/h, which is probably due to the general fluctuation of azimuth angles of the Velodyne lidar, because of the non-uniform rotation speed.

### 3.4. Sunlight

**Figure 13:**
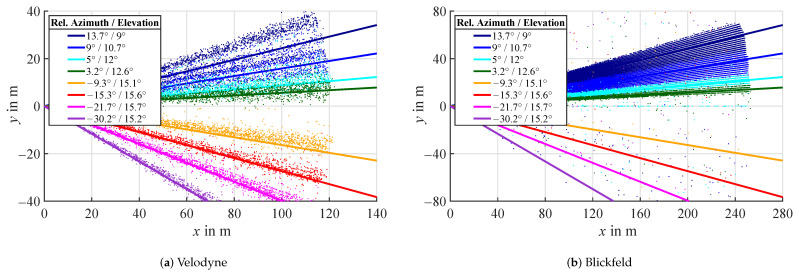
Bird’s eye views of accumulated point clouds over multiple time steps for different positions of the sun. The lines denote the approximate azimuth angle of the sun during the time of the respective measurement relative to the sensor axes.

Sunlight has two different forms of impact on lidar sensors. The first one is ambient light or background radiation as already analyzed by [22] with a Valeo Scala lidar. In this study, no difference in detection intensity due to ambient brightness was detected for any of the three sensors. Therefore, the second influence of sunlight shining directly into the sensor will be the focus. To analyze this effect, measurements were taken on multiple times during sunrise, when the sun travels through the field of view over azimuth and elevation. Both Velodyne and Blickfeld lidars generate atmospheric detections in the direction of the sun, while with the Ouster lidar, no influence in form of detections is measurable. The plots in Figure 13 show bird’s eye views of accumulated point clouds of Velodyne and Blickfeld over multiple time steps for different positions of the sun. The azimuth angles of the sun are taken from a theoretical calculation of the sun position for the time and location of the experiment and denoted as solid lines in the plots with the respective colors of the detection scatter. The angle regions, where the detections appear during the individual measurements, closely correlate to the theoretical solar positions in azimuth and elevation at the measurement time. While the Velodyne lidar generates detections for all measured sun positions, the Blickfeld lidar does so only for the first four. This is due to the sun rising in elevation and surpassing the vertical field of view of the Blickfeld lidar above 17°. The distances to the detections stretch over the entire detection range of the respective sensor. The distance distribution is analyzed further below.

**Figure 14:**
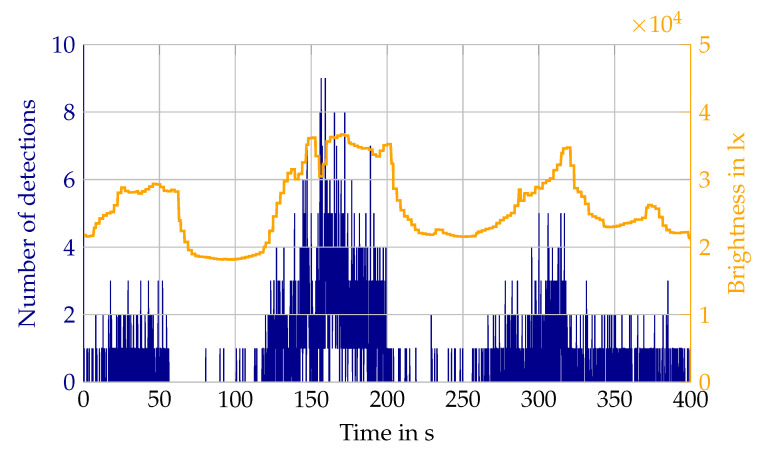
Absolute number of detections in the atmosphere from the Velodyne lidar during sun influence with changing brightness due to clouds.

In order to assess the brightness, at which detections start to appear in the atmosphere, a measurement with varying cloudiness is analyzed. In the measurement in Figure 14 clouds are covering the sun at different time steps, leading to a variation in brightness between 18,000 lx and 36,000 lx. The detection threshold is surpassed at around 20,000 lx in the Velodyne lidar.

**Figure 15:**
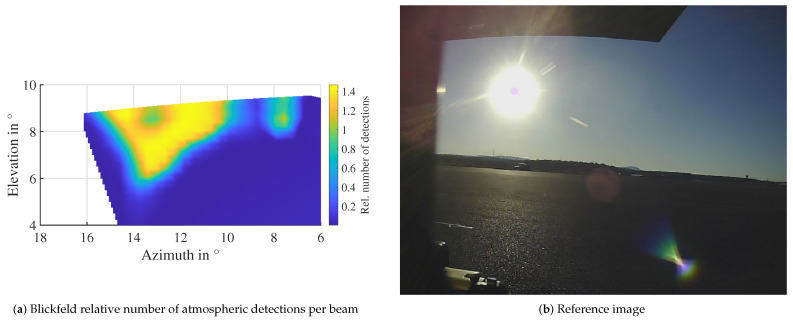
Angle distribution of detections due to direct sunlight influence. A lower detection probability in the center of the sun is visible in the lidar data corresponding to the “corona” visible in the camera image. The approximate sun position relative to the sensor at the time of the measurement was 13.7° azimuth and 9° elevation.

In contrast to precipitation and fog, the detections in the atmosphere do not originate from droplets distributed all around the sensor. The interference originates from a specific combination of azimuth and elevation angles. So, the question is how the detections are distributed over these angles in the point cloud. To analyze this question, the relative number of detections in the atmosphere is plotted over the angles in a mesh. The relative detection count here is the mean number of detections in that beam, averaged over several time steps. An example for the Blickfeld lidar is depicted in Figure 15a. The reference image (Figure 15b) shows a clear sky without interfering clouds. Similar to the camera image where the center of the sun is darker, the number of detections is also lower in the center. A total of 90% of detections are within ±3° in azimuth and elevation from the center of the sun in this measurement. The maximum is reached between 1° and 1.3° from the center.

**Figure 16:**
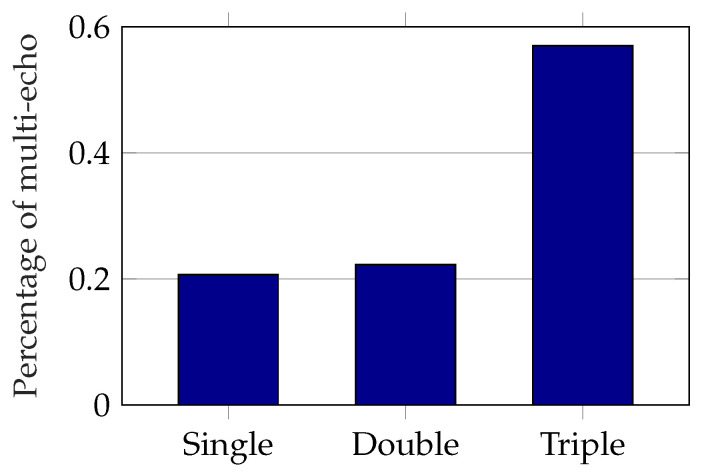
Percentage of multi-echo in detections from sunlight influence of Blickfeld lidar.

As mentioned in previous sections, the Blickfeld lidar is configured with up to three multi-echos per beam and time step. This explains the mean detection numbers per beam being over 1 in Figure 15a. The percentage of single, double, and triple echos per beam detected by the Blickfeld during direct sunlight is depicted in Figure 16. Over half the beams affected by sunlight generate three echos per time step.

**Figure 17:**
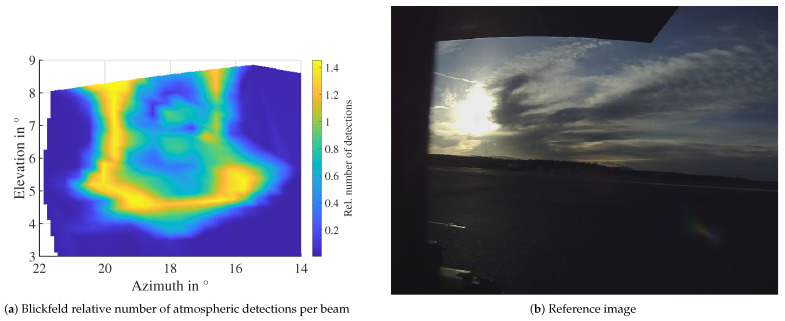
Clouds affect the angle distribution of atmospheric detections due to sunlight. The approximate sun position relative to the sensor at the time of the measurement was 18.1° azimuth and 4.3° elevation.

However, what if the sky is not clear and clouds are partly covering the sun? An example of this case is depicted in Figure 17. With the sun illuminating the clouds and the light is scattered in a bigger angle region, the influence on the sensors differs in terms of elevation and azimuth angle. While the overall angle area remains about the same with ±3°, the maximum values located around a 2° angle distance from the center are further away and the slope towards the maximum rim is much steeper. This means that clouds and their shapes cannot be neglected when simulating the influence of direct sunlight on lidar sensors.

**Figure 18:**
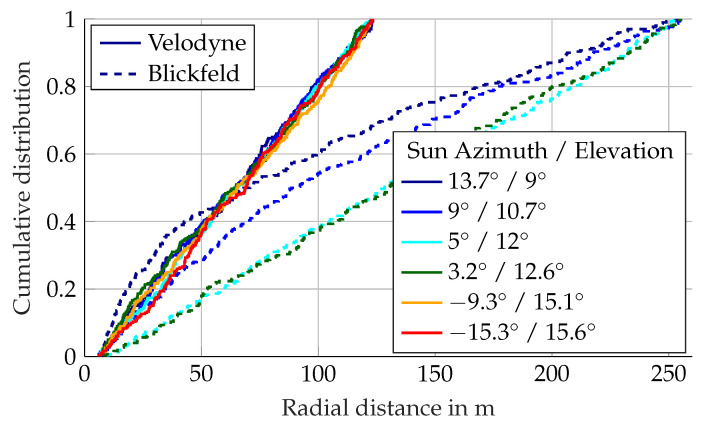
Radial distances of detections in the atmosphere due to direct sun light.

The last question for the influence of direct sunlight on detections in the atmosphere is the distance distribution. Figure 18 shows the distributions in Velodyne and Blickfeld lidars with different sun positions. The detection distances cover the entire respective detection range of the sensors. The Velodyne lidar shows a uniform distribution of detections over the range for all measured sun positions. The Blickfeld lidar has a uniform distribution at 5° and 3.2° azimuth, but at higher azimuth angles, there are more detections closer to the sensor. This is because, in the center of the sun, where the detection probability is lower, detections are closer to the sensor. As the sun rises, this region moves out of the vertical field of view. Hence, the overall detection distances are closer to the sensor during the earlier recordings.

**Figure 19:**
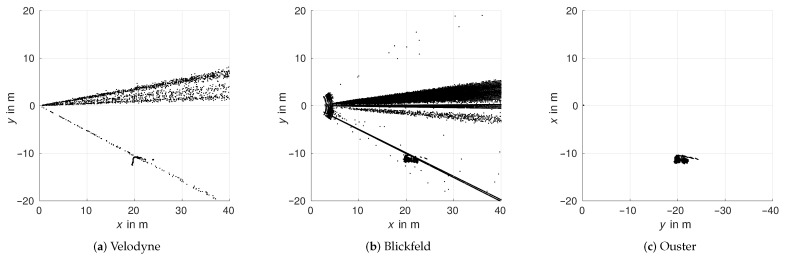
Bird’s eye view over multiple time steps with direct sunlight influence and additional sunlight reflection on vehicle.

Last but not least, there is one more influence by the sun. Sunlight does not only influence the sensor as background radiation or via a direct path from the sun to the sensor. In certain scenarios, the light is reflected by an object in the field of view of the sensor. In the last experiment, a vehicle was placed at approximately 20 m in the x-direction and 10 m in the y-direction in the field of view of the sensors during sunshine and a clear sky shows this phenomenon. Figure 19 depicts the influence on Velodyne, Blickfeld, and Ouster lidars. For Velodyne and Blickfeld lidars, the direct influence of the sun is visible close to 0° azimuth. However, there is also a distinct influence visible from the direction of the vehicle. The Ouster sensor is neither influenced by direct sunlight nor by the reflection off the vehicle.

## 4. Discussion

The overall methodology is based on forming value pairs consisting of one lidar value, such as the relative number of detections in the atmosphere, and one reference value, such as the rain rate in mm/h. These value pairs are aggregated over all measurements of a certain environmental condition to calculate mean values either as an approximation for stochastic modeling or as a reference for model calibration or validation. The following aspects pose a challenge in executing the proposed methodology.

**Figure 20:**
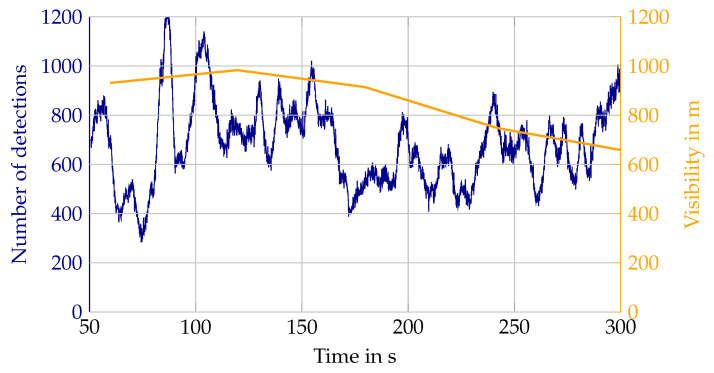
Individual measurement of Blickfeld in fog.

In Figure 20 the absolute number of detections in the atmosphere during a Blickfeld lidar measurement in fog is depicted over time in blue. It is accompanied by the reference measurement of the fog sensor providing the visibility in m for consecutive intervals of one-minute lengths, shown in orange. The values between the measurement points of the fog sensor are interpolated linearly. There are two challenges to make out of this measurement. First, the number of detections varies in a much higher frequency than the output frequency of the fog sensor. Second, even if the time interval of the visibility output was shorter, the problem of inhomogeneity of the fog remains, also depending on wind speed and direction. The fog sensor is oriented the opposite way from the lidar, because of eye-safety reasons, so it cannot capture the actual visibility inside the field of view of the lidar. This leads to greater noise in the value pairs, as was already shown in Figure 4a. The same issues lead to local minima in the detection count on the targets (see Figure 6), because for single-point comparison, the interpolated visibility measurements do not show the true visibility at that point.

**Figure 21:**
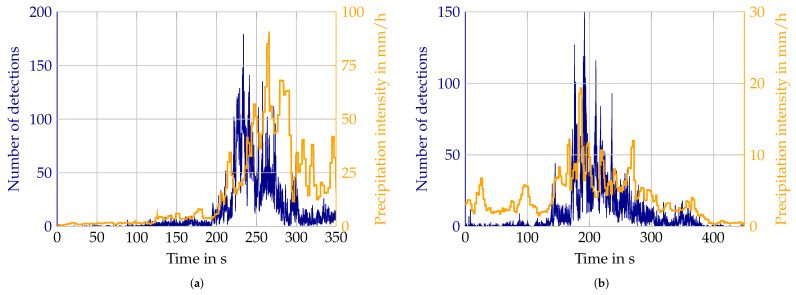
Outliers in Velodyne lidar rain measurements. (**a**) Timing mismatch between Velodyne lidar and reference in rain; (**b**) Mismatch of individual peaks between Velodyne lidar and reference in rain.

The precipitation sensor outputs a mean value of the past 10 s every 3 s and has therefore a higher frequency than the fog sensor. Furthermore, it is not pointed to the opposite side of the sensor fields of view, but upwards and thus is closer to the actual field of view of the lidars. However, mismatches between lidar data and reference still occur. Figure 21a shows a timing difference between the peaks in the number of detections and precipitation intensity. One possible explanation is that rain also is not homogeneous in every case. Wind influence might also play a role. While the precipitation sensor is closer to the field of view, it still does not capture the exact region the lidar is pointing at, so differences might still occur. In Figure 21b the general trends of the curves match, but individual peaks are not aligned. This is possibly due to the averaging over 10 s of the precipitation done by the sensor. This again leads to greater discrepancies in the value pairs aggregated over all measurements.

**Figure 22:**
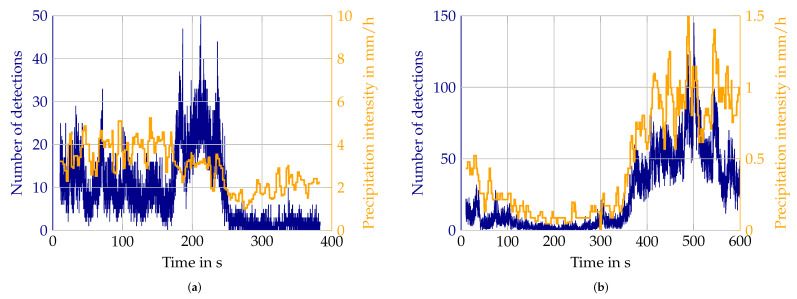
Example of the difference in how well sensor values and reference data match. Additionally, the scale of numbers of detections is quite different. (**a**) Snow measurements, where trends in Velodyne data and reference do not match; (**b**) Snow measurement with matching behavior of Velodyne lidar and reference.

Additionally, in snow, there are mismatches between reference and lidar measurements. While for the most part the trends in both curves fit quite well, Figure 22a shows a counter-example. The higher number of detections between 190 s and 230 s is not reflected in the reference. This has multiple possible explanations. Again, the displacement between the reference sensor and lidar field of view plays a role. However, the snow itself might have changed to a different snowflake size or content of liquid water, which are parameters not captured by the reference sensor. Snow is generally very challenging to measure and is also influenced by the wind, stated in [29]. A fact supporting this hypothesis is a discrepancy in the scale of the number of detections between snow measurements on different days. When comparing the snow measurements of different days depicted in Figure 22, the factor between precipitation intensity and the number of detections in Figure 22b is around 100 and in Figure 22a it is less than 5. This also shows in the aggregated value pairs, as already discussed in Section 3.3.

Despite these examples of differences in sensor data and reference, for the majority of measurements they match quite well. It only becomes an issue in some rapidly changing weather conditions. The big data approach of aggregating all measurements and building median values averages the mismatches out and the methodology is still able to produce expectation values for the impact of all considered environmental conditions.

## 5. Conclusions and Outlook

In this publication, a methodology was introduced to gather real-world measurements of perception sensors in adverse environmental conditions and to analyze the data towards the goal of sensor model development, calibration, and validation. Measurements with three different lidar sensors were taken in a stationary setup over the course of 6 months in rain, snow, fog, and direct sunlight. The data are analyzed by forming value pairs of one lidar value with one reference value each for every time step. These value pairs are aggregated and averaged over all measurements within one environmental condition. This leads to expectation values for detection probability, distance distribution, and attenuation for atmospheric detections on 50% reference targets. It is shown that all four analyzed conditions influence lidar detection data adversely. Especially the need to include direct sunlight influence and cloud formations into lidar simulation models is a valuable insight of this publication. While the values show the same overall trends in the data of the three considered sensors e.g., increasing number of detections for rising precipitation rate, they vary greatly in scale and slope. In particular, the distance distributions of detections in the atmosphere and target detection thresholds of the various sensors are quite diverse. All three sensors operate with the same general sensing principle and in the same wavelength region but seem to have different signal processing pipelines with parameterizations set towards different goals. The Velodyne sensor for example does not produce atmospheric detections during fog while the Ouster sensor performs better in direct sunlight. This entails that accurate physical modeling of environmental effects is not sufficient on its own. The individual sensor peculiarities in hardware and especially in signal processing have to be considered. If they are unknown, they need to be approximated with measurements such as the ones taken within this work. Additionally, the expectations values from the measurements can be used directly to parameterize stochastic sensor models e.g., augmenting clear weather point clouds with atmospheric detections. This will enable the generation of synthetic training data to improve machine learning object detection algorithms in automotive perception systems.

While the overall methodology shows very promising results, there are some points and challenges to be addressed in future work. One, the evaluation will be extended to assess detection intensities and distribution over elevation in future work by the authors. The results will then be applied to stochastically model the lidar behavior in the mentioned conditions. The relative numbers of detection in the atmosphere provide a detection probability per beam. With the additional distance distribution, atmospheric detections can be added to clear condition point clouds. The relative detection count on the targets provides reference values to set the detection threshold during attenuation. For existing physical models, the obtained values enable validation of the model and its calibration by re-simulating the experimental setup and comparing the results. Second, there are some further analysis areas to be addressed in future work with the provided data. While no influence of background radiation on the detection intensity on the targets was identified in this publication, there might be an influence on atmospheric detections during precipitation and fog. This needs to be addressed using the brightness reference measurements in future work. Third, there are some lessons learned from the experiments to be taken into account, if one seeks to repeat these measurements in an improved version. Especially in snow, there seems to be a difference between the reference and the lidar values during various snow events probably due to differences in snowflake size and liquid water content. In rain, there might also be variations in drop size distributions. It is therefore advisable to include a precipitation sensor capable of measuring drop size and snowflake size distributions alongside the precipitation rate. This might also be valuable for the fog measurements. In terms of fog, it would be better to use a more compact sensor, measuring even closer to the sensor’s fields of view without eye-safety restrictions. For both references, the measurement and data output frequencies have to be high enough to accommodate the changes in the environmental conditions. A direct output without averaging filter in a frequency of 1 Hz should suffice for the experience of the authors. Another challenge with the reference is the influence directly on the sensor cover. During the experiments, this was mitigated to some extent with a roof extension over the lidar sensor. However, in strong winds, water and snow still find their way onto the covers. For future measurements it is advisory to place a camera on the station pointed at the sensors to be able to qualitatively assess the state of the sensor covers. Another point to improve the reference is the lidar targets. While the target reflectivity was measured prior to the experiment, it was not validated if the reflectivity stayed the same over the course of the measurements. Although it does not seem like it changed from the intensity measurements of the lidar, reference measurements of the target reflectively in certain intervals throughout the experiments would be beneficial.

Another challenge encountered during the experiments is cross-talk. One additional lidar sensor needed to be excluded from the evaluation due to major issues with cross-talk from the other sensors. Multiple attempts to shield the sensor from the direct impact of the others failed. Not only direct impact is an issue, but also indirect cross-talk e.g., by one sensor illuminating fog surrounding the sensors and the other capturing the signal. There is a general conflict of goals between getting measurements from one sensor without any other influences than from environmental conditions and the goal to compare multiple sensors in these conditions. They should therefore share the same field of view and the same reference targets. Real-world conditions are not easily replicated and these measurements take quite some time and effort, so recording as many sensors as possible at once does make sense, but it entails the possibility of mutual interference of the sensors.

## Data Availability

The dataset recorded for this publication is available open-access at https://www.fzd-datasets.de/weather/, accessed on 13 May 2022.

## Abbreviations

The following abbreviations are used in this manuscript:

CCR	Cube Corner Reflector
CDF	Cumulative Distribution Function
ODD	Operational Design Domain
ROS	Robot Operating System
